# RIG-I-Like Receptor Signaling in Singleton-Merten Syndrome

**DOI:** 10.3389/fgene.2017.00118

**Published:** 2017-09-12

**Authors:** Changming Lu, Mary MacDougall

**Affiliations:** ^1^Institute of Oral Health Research, School of Dentistry, University of Alabama at Birmingham, Birmingham AL, United States; ^2^Faculty of Dentistry, University of British Columbia, Vancouver BC, Canada

**Keywords:** Singleton-Merten syndrome, *IFIH1*, *DDX58*, MDA5, RIG-I

## Abstract

Singleton-Merten syndrome (SMS) is an autosomal dominant, multi-system innate immune disorder characterized by early and severe aortic and valvular calcification, dental and skeletal abnormalities, psoriasis, glaucoma, and other varying clinical findings. Recently we identified a specific gain-of-function mutation in *IFIH1*, interferon induced with helicase C domain 1, segregated with this disease. SMS disease without hallmark dental anomalies, termed atypical SMS, has recently been reported caused by variants in *DDX58*, DEXD/H-box helicase 58. *IFIH1* and *DDX58* encode retinoic acid-inducible gene I (RIG-I)-like receptors family members melanoma differentiation-associated gene 5 and RIG-I, respectively. These cytosolic pattern recognition receptors function in viral RNA detection initiating an innate immune response through independent pathways that promote type I and type III interferon expression and proinflammatory cytokines. In this review, we focus on SMS as an innate immune disorder summarizing clinical features, molecular aspects of the pathogenetic pathway and discussing underlying mechanisms of the disease.

## Singleton-Merten Syndrome

Classic Singleton-Merten syndrome (SMS) is an extremely rare multisystem disorder with a broad phenotypic spectrum associated with variable expressivity. The core features of this disorder include aortic and valvular calcification, dental anomalies, osteopenia or osteoporosis. It was first reported by Singleton and Merten (1973) in two female patients. Gay and Kuhn (1976) described two unrelated patients with clinical and radiological features similar to those of the first cases naming the disorder SMS. In the initial cases of SMS, the dental findings were described as dentin dysplasia. However, Feigenbaum et al. (2013) longitudinally analyzing multiple affected SMS patients broadened the breath of the dental phenotype. Major SMS developmental dental abnormalities now include a delay in primary tooth exfoliation and permanent tooth eruption, truncated root formation mostly in the anterior teeth, early-onset periodontal disease, severe root and alveolar bone resorption that leads to premature tooth loss and an increase rate of dental caries.

More than 40 years since the first documented SMS case, using whole-exon with targeted sequencing, we first reported a gain-of-function missense mutation in *IFIH1* segregating with the disorder (Rutsch et al., 2015). Concurrently, Jang et al. (2015) reported a family affected by glaucoma, aortic calcification and skeletal abnormalities, and another family harboring neither dental anomalies nor aortic calcification, but also suffered from glaucoma and skeletal abnormalities. The authors delineated the condition as atypical SMS since these patients presented with normal dentitions. In addition, a family presenting with varying skin and neurological phenotypes diagnosed as Aicardi-Goutières syndrome (AGS) with overlapping SMS features was reported to have a different heterozygous missense mutation in *IFIH1* determined by whole-exon sequencing (Bursztejn et al., 2015). Pettersson et al. (2017) recently documented two patients, with the same *IFIH1* mutation as our finding, presenting clinical features of both SMS and severe systemic lupus erythematosus (SLE). Very recent report from de Carvalho et al. (2017) revealed another five patients with the clinical phenotype of classic SMS caused by two novel mutations in IFIH1. The clinic features and genetic mutations of classic SMS and atypical SMS are summarized in **Table 1**.

**Table 1:** Summary of clinical features and genetic mutations in classical and atypical Singleton-Merten syndrome (SMS).

	Classical SMS (with dental abnormalities)				Atypical SMS
	Patients (n/N) from	Patients (n/N) from	Patients (n/N) from	Patients (n/N) from	Patients (n/N) from
	Feigenbaum et al. (2013)	de Carvalho et al. (2017)	Bursztejn et al. (2015)	Pettersson et al. (2017)	Jang et al. (2015)
**Clinical features**					
Neurological phenotype	2/11	0/5	2/3	nd	0/11
Lupus phenotype	0/11	0/5	1/3	1/2	0/11
Aortic calcification	10/11	2/4	1/3	1/2	5/7
Cardiac arrhythmia	6/11	nd	nd	nd	0/11
Dental abnormalities	10/11	5/5	1/3	2/2	0/11
Osteopenia	9/10	3/5	nd	1/2	1/8
Acro-osteolysis or tuft erosion of distal phalanx	6/9	5/5	nd	nd	8/8
Wide medullary cavities in the phalanges	9/10	1/5	nd	nd	0/11
Joint deformities	8/9	4/5	1/3	2/2	1/11
Short stature	6/9	3/4	nd	1/2	2/11
Scoliosis	3/10	nd	nd	nd	0/11
Skin abnormalities	8/9	3/5	3/3	1/2	7/11
Glaucoma	5/10	1/5	1/3	1/2	10/11
Subungal calcifications	3/8	nd	nd	nd	0/11
Tendon rupture	6/11	1/5	nd	nd	0/11
Thick neurocranium	8/9	nd	nd	nd	0/11
Distinct facial features	7/7	5/5	nd	nd	0/11
Weakness or hypotonia	8/10	2/5	nd	1/2	0/11
**Genetic mutations**					
MDA5	p.Arg822Gln	p.Thr331Ile p.Thr331Arg	p.Ala489Thr	p.Arg822Gln	
RIG-I					p.Cys268Phe p.Glu373Ala

*n/N, the positive patient number/total examined patient number; nd, undetermined*.

## Gain-of-Function RLR Mutations in SMS

**FIGURE 1:**
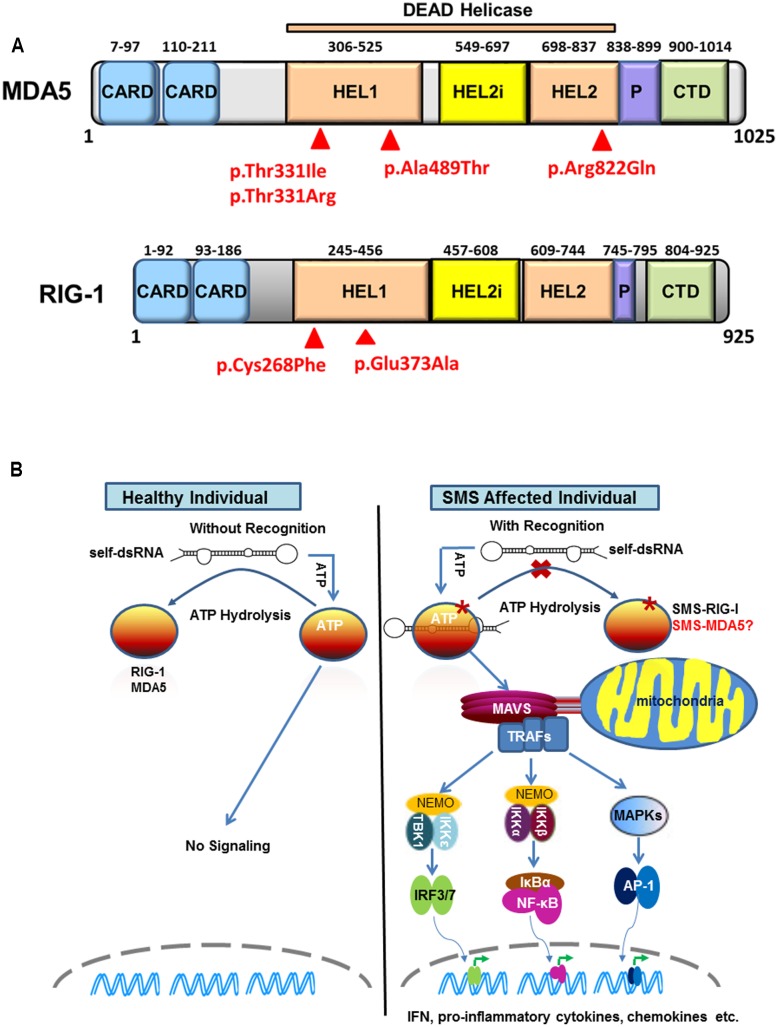
Sustained retinoic acid-inducible gene I (RIG-I)-like receptor signaling in Singleton-Merten syndrome (SMS). **(A)** Structure of melanoma differentiation-associated gene 5 (MDA5) and RIG-I indicating identified SMS mutations in the DEAD helicase domain. MDA5 and RIG-I shared structural features that include: the N-terminal region with two tandem caspase activation and recruitment domains (CARDs); the central DEAD helicase region with HEL1 and HEL2 domains and a helicase-insert domain (HEL2i); the pincer domain; and the C-terminal domain (CTD), also called the regulatory or repressor domain. The most common SMS MDA5 p.Arg822Gln substitution is located in the end of the HEL2 domain. The MDA5 p.Thr331Ile, p.Thr331Arg, p.Ala489Thr and the RIG-I p.Cys268Phe and p.Glu373Ala substitutions associated are located in the HEL1 domain. **(B)** Sustained RIG-I receptor signaling in SMS cells through self-dsRNA recognition. In healthy wild type cells, ATP hydrolysis prevents MDA5 and RIG-I to recognize self-dsRNA thus preventing triggering of signaling. In atypical SMS cells, Glu373Ala mutation (SMS-RIG-I) allows ATP promoted binding of dsRNA to RIG-I, but prevents ATP hydrolysis dependent dissociation of helicase domain from dsRNA, which result in sustained RIG-I signaling and excessive production of IFN, proinflammatory cytokines, chemokines, and others. Similar mechanism may functional related to the MDA5 Ile331, MDA5 Arg331, MDA5 Thr489, and MDA5 Gln822 (SMS-MDA5) identified in classic SMS.

Retinoic acid-inducible gene I (RIG-I)-like receptors (RLRs) are cytoplasmic sensors that recognize the double strands of RNA (dsRNA) of virus and secreted bacterial nucleic acids. So far, three RLR members have been identified: RIG-I, MDA5, and LGP2 (laboratory of genetics and physiology 2). All three of these RLR receptors have similar structures. MDA5 and RIG-I consist of three distinct domains: N-terminal tandem caspase activation and recruitment domains (CARDs), central DExD/H box RNA helicase domain, and C-terminal domain (CTD) (Yoneyama et al., 2004, 2005). The first domain is used to interact with mitochondrial antiviral-signaling adaptor protein (MAVS) to trigger downstream signaling. LGP2 lacks this domain and fails in signaling. The latter two domains are responsible for recognizing viral dsRNA and secreted bacterial nucleic acids (Goubau et al., 2013) (**Figure 1A**).

Structure-function analyses of RIG-I demonstrate its signaling activity is tightly auto-regulated (Saito et al., 2007). In the absence of RNA ligand, RIG-I exists in an auto-inhibited state where the CTD domain folds back to the CARDs, thereby shielding them from the cytosol. With RNA ligand, such as virus infection, dsRNA is initially captured by the CTD followed by co-operative tight binding of ATP and RNA to the helicase domain. The protein undergoes conformational changes to release CARDs from its auto-inhibited state to mediate associations with its adaptor protein MAVS for downstream signaling (Jiang et al., 2011; Kowalinski et al., 2011; Luo et al., 2011). Unlike RIG-I, MDA5 signaling is not regulated by its CTD. After detecting dsRNA, MDA5 uses direct protein–protein contacts to stack along dsRNA in a head to tail arrangement to form a MDA5 filament (Wu et al., 2013). The CARDs decorating the outside of core, MDA5 filament oligomerizes into an elongated structure that activates the signaling adaptor MAVS through CARD–CARD interaction. Upon dsRNA binding to MDA5/RIG-I, the tumor necrosis factor (TNF) receptor-associated factors (TRAFs) are recruited to MAVS to assemble a signaling complex at the mitochondrial outer membranes (West et al., 2011; Xie, 2013). The downstream signaling components are further recruited by TRAFs, leading to the phosphorylation and activation of IRF3 (interferon regulatory factor 3), IRF7, NF-κB (nuclear factor kappa-light-chain-enhancer of activated B cells), and AP-1 (activator protein 1). These transcription factors work cooperatively in the nuclei to induce the expression of interferons (IFNs), proinflammatory cytokines, and other genes that are important in virus clearance.

Using whole-exon sequencing, we identified a common missense mutation, c.2465G > A (p.Arg822Gln) in *IFIH1* in four classic SMS subjects from two families and a simplex case (Rutsch et al., 2015). Sanger sequencing validated the *IFIH1* variant in all 11 affected subjects, but not in unaffected individuals, demonstrating co-segregation with this disease. The p.Arg822Gln mutation is located in the evolutionary conserved motif VI motif of MDA5’s HEL2 domain (**Figure 1A**). Structural and biological studies of MDA5 have revealed that the canonical role of this motif is in ATP hydrolysis (Bamming and Horvath, 2009). Amino acid alterations in this motif are reported to reduce the ATP hydrolysis activity and result in constitutive activation of interferon β (IFN-β). Consistently, over-expression of p.Arg822Gln MDA5 leads to a higher level of *IFN-β* in HEK293T cells compared with normal wild-type MDA5. Moreover, the p.Arg822Gln mutation makes MDA5 hyperactive to polyinosinic-polycytidylic acid [poly (I: C)] with high molecular, a dsRNA analog. Many interferon signature genes are also found to be increased in the whole-blood samples as well as dental enamel organ epithelial cells from SMS patients. These findings demonstrate the gain-of-function mutation of MDA5 in SMS patients. Immunohistochemical staining shows MDA5 is expressed in variety types of cells in major target tissues altered in SMS, although it is not clear if MDA5 expression is increase in SMS patients as compared to healthy individuals.

Jang et al. (2015) described one family affected by atypical SMS and identified a missense mutation (c.1118A > C [p.Glu373Ala]) in *DDX58* that encodes RIG-I. Mutational analysis of *DDX58* in 100 individuals with congenital glaucoma identified another family with the variant c.803G > T, p.Cys268Phe presenting with neither dental anomalies nor aortic calcification but having skeletal abnormalities. The Cys268 and Glu373 residues of RIG-I are located in RIG-I’s helicase domain (**Figure 1A**), belonging to ATP-binding motifs I and II, respectively (Kowalinski et al., 2011). They further demonstrated that Glu373Ala and Cys268Phe mutations led to a gain-of-function of RIG-I. In luciferase assays, elevated amounts of the altered DDX58 structures were associated with significantly enhanced NF-kB reporter gene activity at the basal level, and this activity was further increased by poly(I:C) stimulation. In addition, over-expression of RIG-I mutations sufficiently induced IRF3 phosphorylation and IRF3 dimerization at the basal level. As a result, RIG-I mutations led to increased expression of *IFN-β*, *interferon-stimulated gene 15*, and *chemokine (C-C motif) ligand 5* in both basal and poly (I: C) transfected cells. More importantly, they found that over-expression of RIG-I mutations in human trabecular meshwork (HTM) cells resulted in cytopathic effects and a significant decrease in cell number in comparison with controls. Because HTM cells are responsible for draining the aqueous humor from the eye, the death of HTM cells might represent an underlying mechanism for elevated intraocular pressure leading to glaucoma (Alvarado et al., 1984), one of core manifestations of atypical SMS.

A family with unusual cutaneous features found in AGS and enhanced type I interferon signaling was shown to have a heterozygous gain-of-function *IFIH1* mutation with overlapping features of SMS (Bursztejn et al., 2015) including premature loss of teeth exhibiting root dysplasia. Whole-exon sequencing identified a c.1465G > A, p.Ala489Thr *IFIH1* alteration segregating with the disease. The three affected family members presenting with variable expressivity of neurological and skin features had enhanced type I IFN signature as compared to unaffected individuals. The Ala489 variant, found in the HEL1 domain, resulted in IFN induction not only with poly (I: C) mimicking long dsRNA but also with short dsRNA. In addition, the mutant protein showed impaired ATP hydrolysis and increased stability with the RNA complex as compared to the wild-type MDA5. de Carvalho et al. (2017) recently reported another five individuals with classic SMS phenotypes caused by two novel mutations in *IFIH1* (c.992C.T/p.Thr331Ile or c.992C.G/p.Thr331Arg). All five patients tested demonstrated a remarkably upregulation of IFN-induced transcripts. Both of these variants were associated with increased IFN-β expression in the absence of exogenous dsRNA ligand, consistent with constitutive activation of MDA5. Taken together, SMS is caused by a spectrum of gain-of-function mutations in *IFIH1* or *RIG-1*.

## Potential Pathogenic Mechanisms Underlying SMS

Sustained MDA5 or RIG-I signaling in patients with classic or atypical SMS is possibly due to self-RNA recognition (**Figure 1B**). The critical component of MDA5/RIG-I in defense against virus is their capacity to detect the tiny foreign RNA molecules within the sea of cellular self-RNA. To prevent MDA5/RIG-I reaction to robust self-RNA, the host has developed several protective lines. One is that Adenosine-to-Inosine RNA editing by RNA-specific adenosine deaminase 1 to destabilize duplexes formed from inverted repetitive elements within self-RNA, which prevent MDA5 from sensing these RNA to initiate untended signaling (Heraud-Farlow and Walkley, 2016). Another is that evolution has refined MDA5/RIG-I to kinetically discriminate self/non-self RNA through ATPase activity. Louber et al. (2015) found that MDA5 or RIG-I mutations with low ATP hydrolysis activity exhibited constitutive IFN-β induction but it was diminished when mutations prevent RNA binding, indicating that MDA5/RIG-I signaling can be stimulated by cell-intrinsic RNAs and that the ATPase activity is required to avoid self-activation of RLRs. Lassig et al. (2015) recently provided direct evidence showing that ATPase deficient RIG-I constitutively signals through endogenous RNA and co-purifies with self-RNA. These investigators identified a 60S ribosomal expansion segment as a dominant self-RNA that is stably bound by ATPase deficient RIG-I. By studying the impact of ATP on RIG-I signaling on different RNAs, they provide a model to successfully explain the sustained IFN signaling in atypical SMS. According to this model, in healthy cells, sustained binding of RIG-I to self-RNA containing dsRNA stretches is prevented by ATP hydrolysis. The helicase domain can be sufficiently displaced from self-dsRNA stretches because the CTD does not provide a high affinity tether. SMS mutations in RIG-I allow ATP promoted binding of dsRNA and displacement of the 2 CARD module, but prevent ATP hydrolysis dependent dissociation of helicase domain from dsRNA, which will result in an unintended signaling through self-RNA. Although the native self-RNA for MDA5 has not been identified in classic SMS, the mechanism for sustained MDA5 signaling should be similar to that found in atypical SMS.

RIG-I-like receptors signaling has a broad effect on the innate immune response by inducing type I interferon and numerous inflammatory cytokines and chemokines, which are essential for the host to control the pathogen replication and spreading. However, the excessive production or defective negative regulation of type I IFNs and inflammatory cytokines might be related to a predisposition to inflammatory or autoimmune diseases. Type I IFNs are proteins with pleiotropic functions, such as antiviral, antiproliferative, and immunomodulatory activities. Type 1 interferonopathy (exposure to type 1 interferon too much, for too long or at the wrong time) is reported to be linked with many inflammatory or autoimmune diseases including *IFIH1*-mutation associated AGS and SLE. Although the mechanism for SMS development remains unclear, the excessive type I IFNs, type I interferonopathy, and potential other inflammatory cytokines caused by the gain-of-function mutation of MDA5 or RIG-I is likely one of critical factors for this disease.

Chronic inflammation appears to be the central factor in aberrant vascular calcification in general (Bessueille and Magne, 2015). In SMS patients, endothelial cells and smooth muscle cells in aortic and mitral valves might produce higher proinflammatory cytokine and chemokines due to gain-of-function mutation of MDA5 or RIG-I. Macrophages are then attracted to this area and infiltrate the endothecium, producing more pro-inflammatory cytokines. The local augmentation of inflammatory cytokines, such as interleukin 6 and TNF alpha, in the aortic wall has been shown to activate osteogenic transcriptional factors including SRY-Box 9, runt-related transcription factor 2, msh homeobox 2, and osterix (Al-Aly et al., 2007; Shao et al., 2010).

In contrast to mineralization promotion in soft tissues, chronic inflammation is well known to lead to demineralization in hard tissues (Fardellone et al., 2014). Although the mechanism is mostly unknown, dental anomalies in classic SMS patients are likely in part due to abnormal dentinogenesis and dentin structural integrity caused by the specific *IFIH1* gain-of-function mutation. MDA5 signaling affects dentinogenesis possibly in three ways. First, MDA5 signaling could affect odontoblastic differentiation. We reported that numerous odontogenic markers and differentiation-related genes were dysregulated in SMS patient dental pulp cells as compared to sex- and age-matched control cells (Lu et al., 2014). Among of these genes, alpha-1 type I collagen (COL1A1) was found to be reduced at both the mRNA and protein levels. Type I collagen is composed of two glycine- and proline-rich a1 and one a2 chains that are products of two separate genes, COL1A1 and COL1A2. Type I collagen makes up the majority of dentin extracellular matrix, providing a scaffold for intra- and inter-fibrillar dentin mineralization. Alterations in collagen fibril formation and mineralization can result in dentin structural defects (Myllyharju and Kivirikko, 2001). Therefore, the reduction of COL1A1 expression in SMS dental cells could affect the dentin structural integrity, which is consistent with the dentin dysplasia seen in patients with SMS. Second, MDA5 signaling may affect the cell survival of odontoblasts and their progenitors. MDA5 is reported to induce proapoptotic molecules and regulate cell survival in different ways (Eitz Ferrer et al., 2011; Heutinck et al., 2012; Van et al., 2012; Yang et al., 2013). Our studies show that MDA5 is expressed in the dental pulp cells as well as ameloblasts and odontoblasts (Rutsch et al., 2015). Apoptosis could be induced by elevated MDA5 signaling in these cells due to gain-of-function mutation and/or virus infection. A recent report indicated that RIG-I and MDA5 controlled the cell survival of mesenchymal stem cells from mouse bone marrow (Yang et al., 2013). MDA5 may similarly affect the cell survival of mesenchymal cells in dental pulp where odontoblast progenitors are enriched. Third, MDA5 regulates the expression of matrix metalloproteinase 13 (MMP13) that may contribute to the degradation of the extracellular matrix both internally and externally in the tooth. Previous studies have shown that MDA5 signaling activated by poly (I:C) can induce the expression of MMP13 (Radwan et al., 2013). In agreement with the gain-of-function of MDA5 in SMS patients, MMP13 is found to be dramatically upregulated in SMS dental pulp cells (Lu et al., 2014). MMP-13 is an important collagenase and can degrade the components of the extracellular matrix as well as a variety of substrates such as collagen, gelatin, aggrecan, perlecan, and fibronectin. Furthermore, reports suggest MMP-13 is involved in tooth agenesis and dental caries (Tannure et al., 2012; Antunes et al., 2013). It is conceivable that elevated MMP13 could participate in the pathogenesis of the dental abnormalities found in SMS patients.

Osteoporosis and acro-osteolysis seen in SMS have been reported to associate with enhanced osteoclastogenesis (Ma et al., 2013; Thongchote et al., 2015; Park et al., 2016). Osteoclasts are multinucleated giant cells that disassemble and digest the composite of hydrated protein and mineral of the bone, a process known as bone resorption. Osteoclast formation requires the presence of RANKL (receptor activator of nuclear factor κβ ligand) and M-CSF (Macrophage colony-stimulating factor). Except osteoblasts, activated T, B, and dendritic cells are important source of RANKL. In SMS patients, type 1 IFN is increased due to RLR gain-of-function. Besides the antiviral properties, type I IFN is a key cytokine that links innate and adaptive immunity in response to pathogen infection (Mattei et al., 2010), which may induce the activation of immune cells and increase the basal level of RANKL.

## Conclusion and Future Direction

Here, we have summarized the clinical features of SMS and current literature related to the molecular and genetic aspects of this innate immune disease. Current data from our and another groups indicate that dysregulated inflammation caused by MDA5 or RIG-I gain-of-function mutations contributes to the pathogenesis of SMS.

Several important issues remain to be investigated. First, *IFIH1* has been linked to a number of autoimmune diseases, including AGS, SLE, psoriasis, and diabetes (Nejentsev et al., 2009; Zahn et al., 2011; Enevold et al., 2014; Rice et al., 2014). Why does a single gene function change cause so many different inflammatory or autoimmune diseases with varying phenotypes? Lassig et al. (2015) recently provided direct evidence showing that ATPase deficient RIG-I constitutively signals through endogenous RNA and co-purifies with self-RNA. Is it possible that conformational changes by different amino acid substitutions make MDA5 and RIG-I distinctly active to one or more endogenous dsRNA? Interestingly, p.Ala489Thr mutation seen in the patients with overlapping SMS phenotype impaired the ATP hydrolysis activity of MDA5 distinct from no effect of p.Arg720Gln, p.Arg779His, p.Arg337Gly, p.Arg779Cys, p.Gly495Arg, and p.Asp393Val mutations seen in AGS patients (Rice et al., 2014; Bursztejn et al., 2015). Therefore, it is critical to study whether SMS MDA5 can detect self-RNA in the targeted tissue cells (such as dental cells) and whether mutations in MDA5 and RIG-I affect their recognition of tissue- or cell-specific endogenous ligands.

In addition, the cellular and molecular mechanism for diverse SMS phenotype is largely unknown. Of note, even in the same classic SMS family, there is significant clinical phenotypic variation present in different SMS affected individuals. Moreover, the single p.Arg822Gln mutation in MDA5 can cause different diseases related to SMS and AGS (Buers et al., 2017). This variable expressivity suggests that the existence of other genetic factors, epigenetic factors and/or exogenous influences such as exposure to different pathogens are involved. These factors contribute to the different faces of the same type I interferonopathy spectrum. Future studies aiming at investigating the underlying mechanisms of MDA5 and RIG-I contributions to the pathogenesis of SMS and identifying other factors cross-talking with MDA5 and RIG-I signaling will be critical to develop therapeutic targets of MDA5 or RIG-I for SMS.

## Author Contributions

CL conceived, researched, and wrote the initial draft of the review. MM provided additional content, design of the figures, and edited the final version.

## Conflict of Interest Statement

The authors declare that the research was conducted in the absence of any commercial or financial relationships that could be construed as a potential conflict of interest.
